# Odds and Predictors of Monozygotic Twinning in a Multicentre Cohort of 25,794 IVF Cycles

**DOI:** 10.3390/jcm12072593

**Published:** 2023-03-30

**Authors:** Nikit Kadam, George Woodhead, Louise Kellam, Alison Campbell, Kanna Jayaprakasan

**Affiliations:** 1 Derby Fertility Unit, University Hospitals of Deby and Burton, Derby DE22 3NE, UK; 2 Care Fertility, Nottingham NG8 6PZ, UK

**Keywords:** monozygotic twinning, assisted reproduction, blastocyst transfer, IVF, multiple pregnancy

## Abstract

The rate of monozygotic twinning (MZT) has seen a gradual increase in recent years. Numerous parameters involved in ART procedures are blamed for this surge, even though the exact explanation is as yet unknown. Our study’s objectives were to determine the risk variables for monozygotic twinning after ART and to estimate their prevalence. We examined 25,794 IVF cycles for the incidence of monozygotic twinning in this observational analysis. Our study, which was carried out across seven tertiary IVF centres over the course of four years, found an overall MZT rate of 0.37% per embryo transfer procedure and 0.88% of all pregnancies. Monozygotic twinning was more commonly seen in fresh single-embryo transfer (SET) and blastocyst transfer cycles. Larger multicentre studies are needed to explore the potential risk variables.

## 1. Introduction

Monozygotic twining (MZT) is rare and has an incidence of 0.4% in naturally conceived pregnancies [1]. The exact mechanism of MZT in spontaneously conceived pregnancies remains unclear. Advances in medical sciences have made it possible for infertile couples to achieve parenthood through in vitro fertilisation (IVF) procedures. While one of the greatest risks with IVF is multiple pregnancies, the move towards single-embryo transfers (SETs) worldwide has significantly reduced the prevalence of multiple pregnancies [2]. However, SET does not eliminate the risk of multiple pregnancies. MZT can occur after SETs and the incidence of MZT after IVF procedures is significantly higher than in natural conceptions [3]. Multiple gestations with MZT carry significant risks to the foetus and the mother. In addition to the well-acknowledged multiple pregnancy risks, including preterm birth, pre-eclampsia, and low-birth-weight babies consequently raising perinatal mortality and morbidity, MZT poses specific risks, such as twin–twin transfusion syndrome (TTTS), anaemia–polycythaemia syndrome, twin reversed arterial perfusion (TRAP), and umbilical cord accidents [4].

Studies have reported the rate of MZT as about 1.5% following IVF [5,6]. While the exact cause of increased prevalence of MZT after IVF is unclear, a few systematic reviews and meta-analysis have been published to address this [3,4,7]. The possible factors reported include maternal age, extended blastocyst culture and embryo culture conditions [6,8,9]. Some other risk factors, such as zona pellucida manipulation methods used in intracytoplasmic sperm injection (ICSI), assisted hatching, and embryo biopsy for preimplantation genetic testing (PGT), have also been reported to increase the rate of multiple gestation [10,11,12].

Most studies conducted to estimate prevalence and risk factors were retrospective, and the data on MZT after double-embryo transfers are limited. In this observational study, we aimed to estimate the prevalence of MZT and determine the probable risk factors contributing to MZT in a prospectively collected database of a large cohort of women undergoing single-embryo transfer (SET) and double-embryo transfer (DET) following IVF.

## 2. Materials and Methods

Study population: Women of all ages who had undergone IVF/ICSI with fresh and frozen embryo transfer culminating in SET and DET were included. The women’s BMI (body mass index) was less than 35 kg/m^2^. This study consisted of all consecutive IVF treatment cycles across seven tertiary fertility centres in the UK. Data were collected and analyzed over a period of four years from 2013 to 2017.

Clinical protocol and outcome: All women underwent controlled ovarian stimulation protocols using standard antagonist or long agonist protocol depending on clinician preference. The antagonist protocol involved commencing daily gonadotropin injections (recombinant FSH or human menopausal gonadotropins) from day 2 of the menstrual cycle with adding daily subcutaneous injection of 0.25 mg of Cetrotide from day 5 of gonadotropin stimulation. Long agonist protocol involved downregulation using 0.5 mg of buserelin per day, commenced in the mid-luteal phase of the menstrual cycle. Two weeks later, following confirmation of ovarian suppression through finding of an endometrial thickness of <5 mm and no ovarian activity on ultrasound, oestradiol levels were confirmed to be less than 200 pmol/L. Subsequently gonadotropin stimulation were commenced. The dose of gonadotropins ranged between 150 and 450 IU/day, depending on women’s age, ovarian reserve and previous treatment characteristics. The ovarian response was monitored daily by serial transvaginal ultrasound +/− oestradiol measurements on day 6–7 of gonadotropin stimulation and the dose was adjusted depending on the response. Human chorionic gonadotropin (hCG) was administered when there were three follicles of 17 mm or more in diameter and egg retrieval performed 36 h later. IVF or ICSI was subsequently performed. Single or double embryo was transferred at cleavage stage (day 2 or 3) or at blastocyst stage (day 5), as set out by the Human Fertility Embryology Authority (HFEA) policy. Most women had single embryo at blastocyst stage. Luteal support with vaginal progesterone 200 mg three times a day was commenced on the second day after egg collection.

For frozen embryo transfer (FET) cycles, endometrial preparation and luteal support were provided using hormonal preparations of oestradiol and progesterone. Endometrial preparation was started on day 1 of the cycle with oral oestrogen 2 mg three times a day. An ultrasound scan was conducted to ascertain endometrial thickness after 10–12 days. On 15 + 2 days, vaginal progesterone supplementation with progesterone pessaries 400 mg twice daily was commenced once endometrial thickness reached 7 mm. Embryo transfer with one or two embryos was scheduled for day 20 + 2 while oestradiol was maintained at the same dose. Women were advised to abstain from sexual intercourse during the treatment in order to avoid the possibility of concurrent natural conception. Urine pregnancy test was done 12–14 days after embryo transfer. Routine transvaginal ultrasound was performed during the 6th or 7th week after embryo transfer for women who conceived after fresh IVF or FET treatment. The diagnosis of monozygotic pregnancy was made on the visualisation of two foetal hearts in cases of SET and three or more in DET cases.

Statistical Analysis: The primary outcome measure was the incidence of MZT. Relative risk (RR) and 95% confidence intervals (CIs) were calculated. Logistic regression analysis was used to assess the effect of different variables on the chances of MZT occurrence. A *p*-value < 0.05 was considered significant. Analyses were conducted using Statistical Package for Social Sciences software (SPSS Version 26, IBM Corp., Armonk, NY, USA).

## 3. Results

The number of cycles that met the inclusion criteria were 25,794, of which 16,845 were SET and 8949 DET. The mean (± standard deviation) age of the study population was 36.0 (±5.1) years. Out of the 25,794 IVF cycles, 96 pairs of MZT were identified, resulting in and MZT rate of 0.37% (96/25794) per ET and 0.88% (96/10867) of all the pregnancies. The effect of SET and DET on the rate of MZT was analyzed.

### 3.1. SET Cycles

The mean (±standard deviation) age of women with SET was 36.1 (±5.1) years. SET was performed in 16,845 cycles, and 82 cycles developed MZT (0.47% per ET). Table 1 demonstrates the relationship between SET cycles and MZT rate. A statistically significant higher MZT rate was found after blastocyst transfer compared with cleavage embryo transfer (0.58% vs. 0.1%; *p* < 0.05). The MZT rate of all pregnancies was 1.25% for blastocyst transfers and 0.39% for cleavage-stage transfers (OR 3.21, 1.01–10.22; *p* < 0.5). On subgroup analysis, a significant difference in MZT rate between blastocyst and cleavage stage transfers was noted only for fresh embryo transfers (OR 8.4, 1.16–60.9) and not for frozen transfers (OR 0.65, 0.16–2.67). We also studied the relationship between IVF and ICSI and the incidence of MZT. The rate of MZT was similar in conventional IVF and ICSI cycles (0.53% vs. 0.49% *p* = 0.93; OR 1.03, 0.58–1.83). When comparing fresh and frozen SETs, the MZT rates were similar (1.11% vs. 1.23 *p* = 0.66; OR 0.9, 0.59–1.4). The mean age of women with MZT was younger than those with singleton pregnancies (36.1 ± 5.1 vs. 34.9 ± 4.6; *p* = 0.05). However, on regression analysis, age was a significant influencing factor for MZT (odds ratio (95% CI) 0.954 (0.912–0.998); *p* = 0.04).

### 3.2. DET Cycles

In sum, 8949 cycles had DET, of which MZT was seen in 14 cycles (0.16%). Our results showed that the effect of embryo culture length and fresh and frozen ET on MZT in DET cycles was statically insignificant. Similarly, no difference was found when we compared the incidence of MZT in IVF and ICSI cycles (see Table 2).

## 4. Discussion

The data from this multicentre study involving 25,794 cycles indicate an MZT rate of 1.16% and 0.37% of all pregnancies after SET and DET, respectively. The corresponding rates of MZT were 0.49% and 0.16% per ET after SET and DET. The incidence of MZT was higher with patients having a fresh blastocyst SET (1.25%) than with those having fresh cleavage-stage SET (0.39%). A significantly higher MZT rate was also seen in blastocyst SET compared with blastocyst DET, although this could potentially be due to MZT detection limitations in the DET group. The study shows the strongest predictive factor for MZT is a fresh SET, and the lowest odds were with frozen DET. Younger women are marginally more at risk of MZT after SET. This is one of the largest studies published to date on this topic, and the data from this study are helpful in counselling women and couples on their risk of twinning rate, particularly after single-embryo transfer.

The mechanism of MZT remains poorly understood and continues to be an enigma. One of the important ART parameters that potentially governs the rate of MZT is the embryonic stage at which ET is performed. Several systematic reviews and meta-analyses have analyzed this association demonstrating increased rate of MZT after blastocyst transfer.

One such meta-analysis [4] in 2018 was a review of 42 studies that found that MZT numbers were significantly higher after blastocyst transfer than that with cleavage-stage transfer. The question as to why the rate of MZT is higher after blastocyst transfer is a matter of debate. Many theories could provide an explanation without coming to a consensus. Prolonged in vitro culture and increased sensitivity to change in temperature and pH during culture are implicated as possible reasons [13]. Further, high glucose levels in the culture media can lead to generation of free radicals, resulting in increased apoptosis, which in turn culminates into the splitting of the inner cell mass. Another proposed theory is low levels of calcium leading to destabilisation and division of inner cell mass [7]. Changes to the ZP during longer-term culture have also been postulated to influence the likelihood of MZT. Blastocyst hatching, as observed in vitro, may be different in vivo.

According to our data, the overall incidence of MZT was similar in fresh and frozen SET cycles. The results from our study are different to the retrospective study published in 2020 [14], which looked into the effect of fresh and frozen ET on the incidence of MZT. Contradictorily to the findings of our study, Knopman et al. and Nakasuji et al. reported a lower incidence with fresh ET. A study published in 2016 [5] proposed that change in the uterine environment after hyperstimulation was attributable to high incidence of MZT following fresh transfers. Nonetheless, the relationship between fresh versus frozen ET and MZT remains poorly understood.

It has been hypothesised that zona pellucida manipulation methods (ICSI and artificial hatching) have a positive influence on zygotic splitting, thus increasing the incidence of MZT. The opening in the zona pellucida caused by these methods results in herniation and splitting of inner cell mass. Although some investigations found an increased association between MZT rate and manipulation methods [3,5], many other studies [1,15] refute this finding. In our study, we did not observe any difference in MZT rates between IVF and ICSI cycles. On the whole, due to the extremely low occurrence of MZT, there is no clear consensus on the influence of IVF versus ICSI procedures on twinning rates. Further research into different aspects of micromanipulation methods and their association with MZT rate is warranted.

One of the key strengths of our study was inclusion of a large cohort of treatment cycles across seven tertiary centres in the UK, which enabled us to collate the relevant data. The prospectively collected data and unselected population in this study limit any selection bias. The lack of utilisation of ultrasound criteria to identify and confirm MZT at 6–7 weeks in pregnancies following DET is one of the limitations of our study. The gold-standard method to detect MZT would be genetic testing; however, the high cost of genetic testing precludes this option. Secondly, other parameters of ART procedures, such as embryo quality, duration of treatment, type of stimulation protocol, and cause of infertility, which could have an effect on MZT rate, were not analyzed in our study.

## 5. Conclusions

MZT following SET and DET was 1.16% and 0.37% of all pregnancies and 0.47% and 0.16% per ET, respectively, with its odds being highest with fresh blastocyst SETs and lowest with frozen cleavage-stage DETs. Younger women are marginally more at risk of MZT after SET. Elective SET does not eliminate the risk of multiple pregnancies, but is currently the best strategy to lower the multiple-pregnancy rates in ART treatment cycles. Fertilisation technique was found to have less impact on the incidence of MZT. Couples undergoing IVF should be informed of a small chance of occurrence of MZT pregnancy even after SET. Nonetheless, the precise aetiology and incidence of MZT remains uncertain. Larger prospective studies are warranted in order to understand the mechanism underpinning MZT and ART, which would help fertility centres to conceptualise guidelines to improve clinical outcomes.

## Figures and Tables

**Table 1:** SET cycles and MZT rate.

	Total ET	MZT	Not MZT	Total Clinical Pregnancy	CPR per ET (%)	MZT per ET (%)	MZT per all Pregnancies (%)	RR (95% CI)	*p* Value
All SET	16,845	82	7012	7094	42.1	0.49	1.16		
CSE	3145	3	762	765	24.3	0.1	0.39	Blast vs. CSE	
Blastocyst	13,700	79	6250	6329	46.2	0.58	1.25	3.2 (1.01–10.1)	<0.05
Fresh ET	9739	49	4355	4404	45.22	0.50	1.11		
CSE	2590	1	656	657	25.37	0.04	0.15	Blast vs. CSE	
Blastocyst	7149	48	3699	3747	52.41	0.67	1.28	8.4 (1.16–60.9)	<0.05
FET	7106	33	2657	2690	37.86	0.46	1.23		
CSE	555	2	106	108	19.46	0.36	1.85	Blast vs. CSE	
Blastocyst	6551	31	2551	2582	39.41	0.47	1.20	0.65 (0.16–2.67)	0.55
IVF Fresh	3394	18	1574	1592	46.91	0.53	1.13		
CSE	816	0	204	204	25.00	0.00	0.00	IVF vs. ICSI	
Blastocyst	2578	18	1370	1388	53.84	0.70	1.30	(over all)	
ICSI Fresh	6345	31	2781	2812	44.32	0.49	1.10	1.03 (0.58–1.83)	0.93
CSE	1774	1	452	453	25.54	0.06	0.22		
Blastocyst	4571	30	2329	2359	51.61	0.66	1.27		

SET—single-embryo transfer, MZT—monozygotic twinning rate, CSE—cleavage-stage embryo, FET—frozen embryo transfer.

**Table 2:** DET cycles and MZT rate.

	Total ET	HOMP	Not HOMP	Total Clinical Pregnancy	CPR per ET (%)	MZT per ET (%)	MZT per All Pregnancies (%)	RR (95% CI)	*p* Value
All DET	8949	14	3759	3773	42.16	0.16	0.37		
CSE	4361	5	1500	1505	34.51	0.11	0.33	Blast vs. CSE	
Blastocyst	4588	9	2259	2268	49.43	0.20	0.40	1.19 (0.4–3.56)	0.75
Fresh ET	6867	10	2825	2835	41.28	0.15	0.35		
CSE	3873	4	1349	1353	34.93	0.10	0.30	Blast vs. CSE	
Blastocyst	2994	6	1476	1482	49.50	0.20	0.40	1.37 (0.39–4.84)	0.63
FET	2082	4	934	938	45.05	0.19	0.43		
CSE	488	1	151	152	31.15	0.20	0.66	Blast vs. CSE	
Blastocyst	1594	3	783	786	49.31	0.19	0.38	0.58 (0.06–5.5)	0.63
IVF Fresh	2500	6	1081	1087	43.48	0.24	0.55		
CSE	1400	2	506	508	36.29	0.14	0.39	IVF vs. ICSI	
Blastocyst	1100	4	575	579	52.64	0.36	0.69	(over all)	
ICSI Fresh	4367	4	1744	1748	40.03	0.09	0.23	2.41 (0.68–8.5)	0.17
CSE	2743	2	843	845	30.81	0.07	0.24		
Blastocyst	1894	2	901	903	47.68	0.11	0.22		

DET—double-embryo transfer, MZT—monozygotic twinning rate, CSE—cleavage-stage embryo, FET—frozen embryo transfer.

## Data Availability

The authors confirm that the data supporting the findings are available from the corresponding author upon reasonable request.
